# Supplementary material for: Collaborative mapping: Perceived environmental risk of leptospirosis in Urban communities in Salvador, Brazil
Source: PLOS Glob Public Health. 2026 Apr 9;6(4):e0006113. doi: 10.1371/journal.pgph.0006113 (PMC13065017; doi:10.1371/journal.pgph.0006113)
Supplement: S1 Appendix — (DOCX) [file pgph.0006113.s001.docx]

**Supplementary material I**


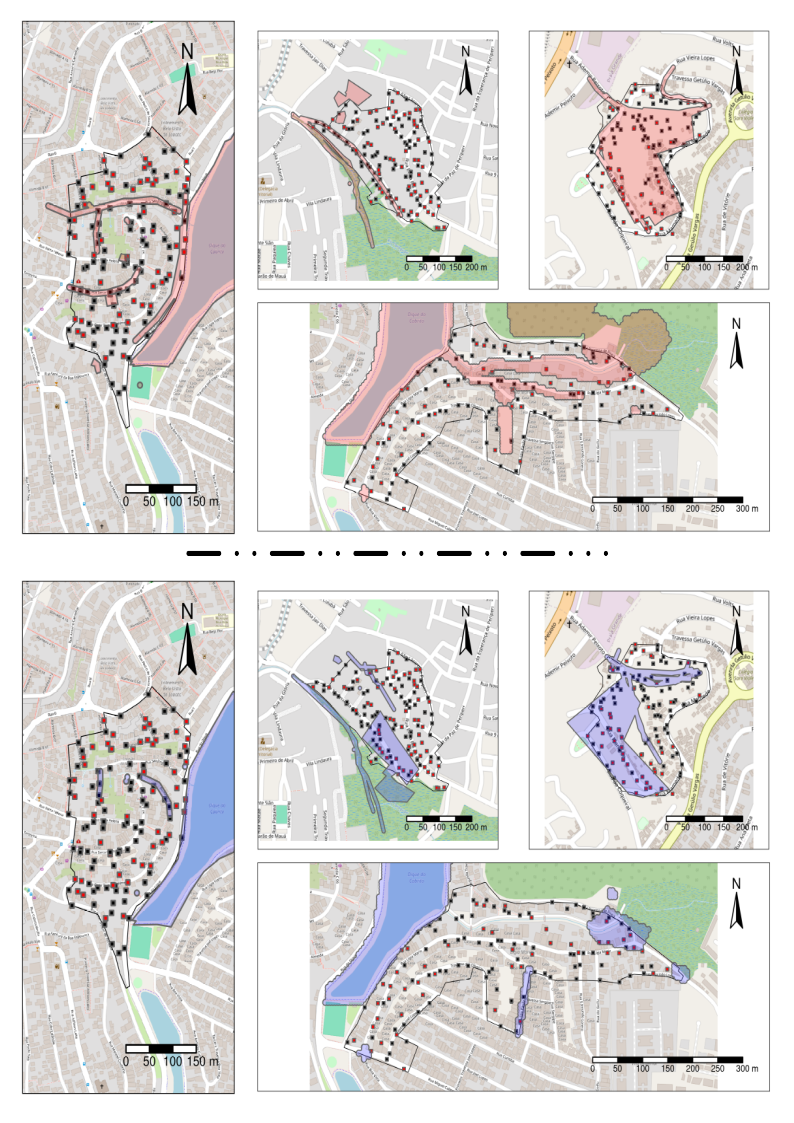
**Fig SI A** Locations of rat traps and track plates (red: positive, black: negative) with respect to areas perceived as high risk (red, top panel) and low risk (blue, bottom panel) for rat presence


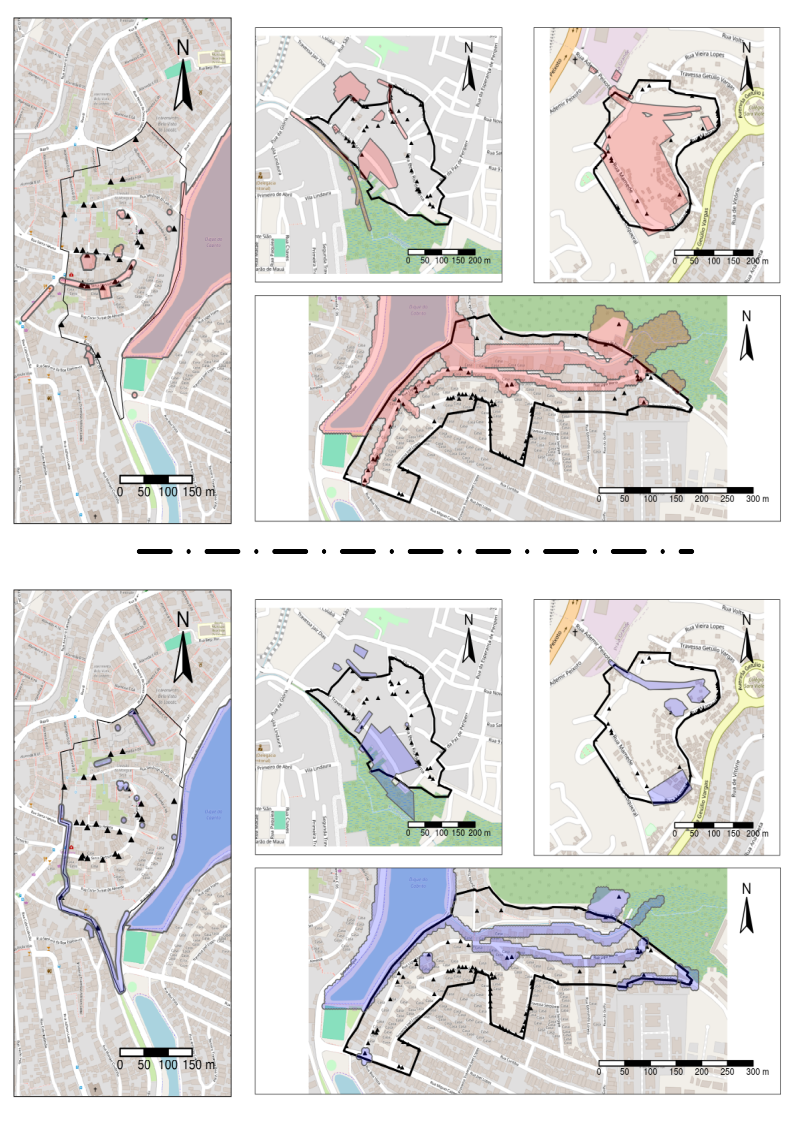
**Fig SI B** Locations of rubbish piles (black triangles) with respect to areas perceived as high risk (red, top panel) and low risk (blue, bottom panel) for rubbish
