# Supplementary material for: Collaborative mapping: Perceived environmental risk of leptospirosis in Urban communities in Salvador, Brazil
Source: PLOS Glob Public Health. 2026 Apr 9;6(4):e0006113. doi: 10.1371/journal.pgph.0006113 (PMC13065017; doi:10.1371/journal.pgph.0006113)
Supplement: S2 Appendix — (DOCX) [file pgph.0006113.s002.docx]

**Supplementary material II**

| 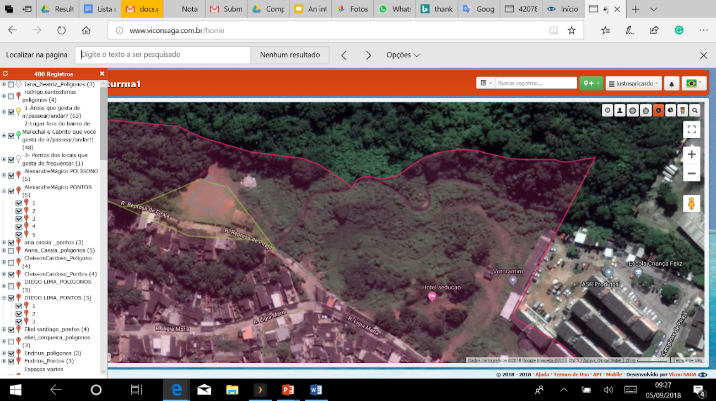  **A)** |
| --- |
| 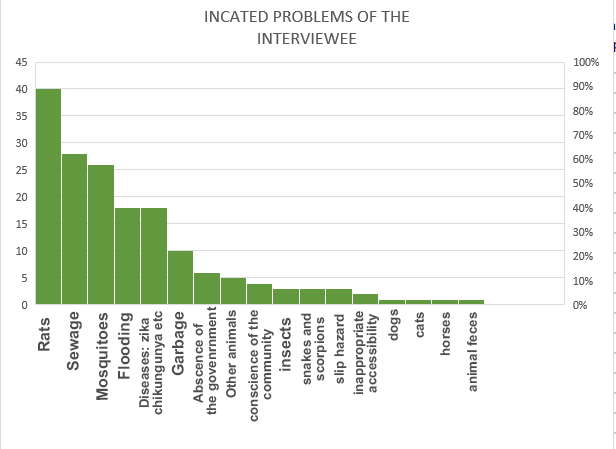  **B)** |
| 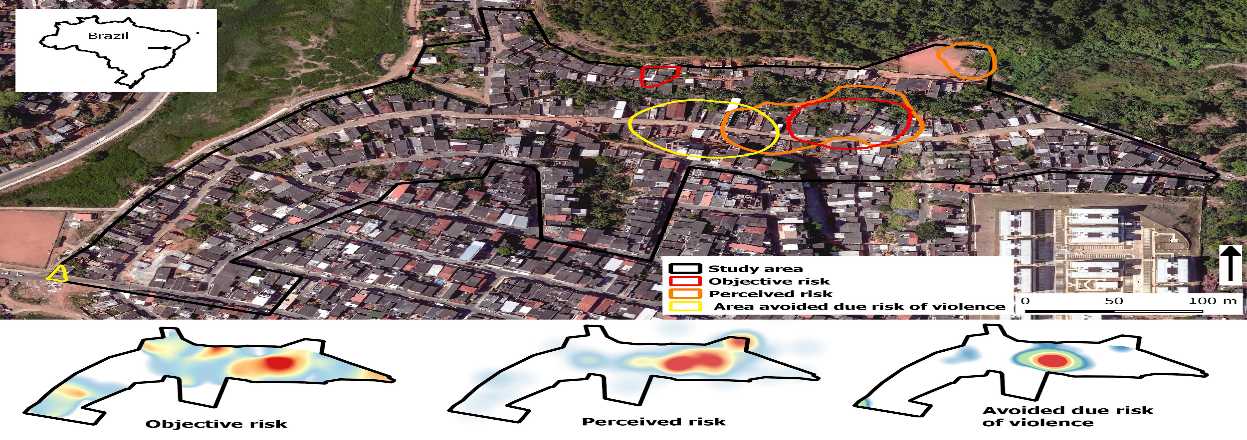  **C)** |

**Fig SII A** Panel showing results presented to communities and public bodies: A) WebMap environment in vicon SAGA showing the specific location of problems indicated by residents; (B) Graphic with categories of problems identified as health risks in the neighborhood; (C) Map of concentration of places indicated as health risks, of people with positive serology for leptospirosis and of places indicated as violent in the neighborhood

After the collaborative mapping stages, graphics, web maps, and thematic maps relating to the results of the collaborative mapping were produced. These materials were prepared in an accessible language to communicate with the population and public agencies to disseminate the findings, promote discussions in the associations involved, and raise awareness among institutional partners. This approach aimed to bring public agencies closer to the communities, create job opportunities for young people, and encourage necessary interventions. The meetings with public agencies focused on social areas, urban maintenance and cleaning, technology, and urban furniture.

The ViconSAGA [21] platform was used to create the WebMap. The young people actively participated in the process, digitizing points, lines, and polygons indicated by residents during the On-field survey phase of the collaborative mapping. These elements were identified as posing a health risk in the neighborhoods, and descriptions of these elements and photos were included. The platform allows any user registered in the web project to access this information for consultation.

In addition to the web map, the research team produced a Kernel Concentration Map, which maps locations indicated as presenting a health risk (subjective risk) and the distribution of people with positive serology for leptospirosis (objective risk). The points and centroids of polygons and lines indicated as presenting a health risk were processed to produce the Kernel map related to the concentration of locations indicated as presenting a health risk. These elements were weighted according to the level of risk attributed by the participants (1 to 3) and with a radius of 50 meters. The Kernel concentration of people with positive serology for leptospirosis was generated from the points of the households, considering the number of positive people as a weight and using a radius of 50 meters. In addition, the points indicated by the young people as presenting violence were processed to generate a Kernel concentration, also with a radius of 50 meters. Subsequently, we created a summary map that integrated the previously described Kernel concentration analyses and the overlapping polygons of locations of objective risk for leptospirosis, subjective risk, and violence. The polygons were created from the last quartile of isolines of the Kernel analyses. In addition to these maps, graphics were produced with the subjective risk categories indicated by residents for each neighborhood. The thematic maps and graphs were created, respectively, in Qgis 2.18 and Excel software.

*Details about the phase* *sharing with communities and public service provider*

The public companies in the city of Salvador visited promoted several educational and social interventions in the communities studied (Figure S2). Among the actions, the following stand out: preparing young people for university entrance exams, artistic interventions with graffiti, implementing a community garden, and creating job opportunities for two young people from the community in public companies. One of these young people reached the position of advisor of the Urban Development Company (DESAL), acting as the project coordinator at the institution. In addition, garbage was removed from a chronic accumulation site, where years of useless waste had been deposited by the population, and a collection box was installed in one of the four participating neighborhoods. Furthermore, the participation of young people in public consultations influenced the inclusion of objectives in the Innovation Law of the city of Salvador, prioritizing actions for black men and women from informal neighborhoods. It was observed, however, that only young people, associations, and communities from two of the four neighborhoods involved were included in actions in partnership with public bodies after the collaborative mapping activities.
