# Supplementary material for: Collaborative mapping: Perceived environmental risk of leptospirosis in Urban communities in Salvador, Brazil
Source: PLOS Glob Public Health. 2026 Apr 9;6(4):e0006113. doi: 10.1371/journal.pgph.0006113 (PMC13065017; doi:10.1371/journal.pgph.0006113)
Supplement: Attachment — Submitted filename: Response to reviewers_Updated.docx [file pgph.0006113.s004.docx]

Pablo Ruiz Cuenca
Lancaster University Medical School
Lancaster
United Kingdom
LA1 4YW
p.ruizcuenca@lancaster.ac.uk

**To:**

**Editors,**

**PLOS Global Public Health**

**Dear Editors**

We thank the editors and the reviewers for their valuable comments and suggestions on the manuscript.

We have carefully considered and responded to all comments, making the appropriate modifications in the manuscript. Our responses refer to line numbers in the new manuscript version (Revised Manuscript with Track Changes).

We believe that the manuscript is now suitable for publication in PLOS Global Public Health.

Sincerely,

Pablo Ruiz Cuenca
PhD Researcher
Lancaster University

**RESPONSE TO REVIEWERS’ COMMENTS**

**Reviewer #1:** Dear Authors, the manuscript is well written. However, there is a lack of method description. The results can do more in this analysis. I have some comments below.

**Comment 1. Abstract: Please provide the full name of four areas.**

**Response:** Thank you for your comment. We have included the full names of the four areas in the study summary (lines 32-33).

**Comment 2. Serological data: It is unclear how to define positive cases. How many serovars were tested? For the positive participants, were they positive for at least one serovar or something else?**

**Response:** We appreciate the reviewer’s insightful comment and the opportunity to clarify this point. In our study, samples were tested against a panel of seven Leptospira strains, including five international reference strains and two local clinical isolates. The tested strains represented the following serogroups: *L. kirschneri* Cynopteri (strain 3522C) and Grippotyphosa (strain Duyster); *L. interrogans* Canicola (strain H. Utrecht IV), Autumnalis (strain Akiyami A), and Icterohaemorrhagiae (serovar Copenhageni, strain Fiocruz L1-130); *L. borgpetersenii Ballum* (strain MUS 127); and L*. santarosai Shermani* (strain LV3954).

Participants were considered seropositive if ≥50% agglutination was observed at a titer of ≥1:50 for at least one of the tested serovars. Samples reactive at a 1:100 dilution were further titrated in serial twofold dilutions. Individuals reactive to one or more serogroups — including mixed reactions - were classified as positive cases.

Although the MAT panel included seven antigens, the serogroups Icterohaemorrhagiae and Cynopteri accounted for the majority of positive reactions, consistent with previous studies in the region (Khalil [1]; De Oliveira [20]). The manuscript has been updated to accurately describe the full panel used and the definition of positivity (lines 115–131).

**Comment 3. Phase 2 Field surveys: How do the authors define the 244 residents (or participants)?**

**Response:** Thank you for your comment and the opportunity to clarify this. To define the participants in our study, we used a census sample from the serological survey conducted in the study areas and approached those who were eligible to participate. Eligible residents were individuals aged >18 years or older, who slept three nights per week within the study area, were able to provide written informed consent, and were considered heads of household. We included these details in the manuscript (lines 150-153).

**Comment 4.** **To understand the survey results, please provide the questionnaire.**

**Response:** Thank you for your comment. The questionnaire is available as an appendix in the supplementary materials.

**Comment 5. Lines: 159-164, It is unclear how to define the type and level of risk as shown in Table 3.**

**Response:** Thank you for your comment and the opportunity to clarify this. The definition of the perceived risk type in our study stemmed from an open-ended question posed to participants and subsequent analysis of the content of the response categories. To define the risk level, studies that used dissatisfaction scales in their methodology, such as the Likert scale, were consulted. However, for this study, our research team considered, after discussion with experts (Ecologist, Epidemiologist, and Veterinarian) and young people, that a direct question to participants with a minimum number of categories would be more effective, based on the characteristics of the population and the context of the areas, aiming not to prolong the interview time, which could cause demotivation in the responses and possible biases. Thus, by consensus, we defined the categories "Low," "Medium," and "High" to represent the level of risk indicated by the participants for the problem reported in their communities.

We have included a summary of these details in the manuscript (lines 177-179). Please see below:

“We developed these categories of risk through discussions and consensus with experts in the fields of ecology, epidemiology, veterinary medicine, and young people from the communities.”

**Comment 6. Line: 181, What do the authors mean by eligible residents?**

**Response:** Thank you for your question and the opportunity to clarify this information. Eligible residents comprised all residents recruited for our research based on our inclusion criteria, which were mentioned in lines 152-154. Please see below:

“Individuals who were aged >18 years old, slept three nights per week within the study area, were able to provide written informed consent, and were considered heads of household were asked to complete a questionnaire.”

For clarification on who the eligible residents are, we have included additional information in the methodology section (lines 150-153).

**Comment 7. For the environmental characteristics, since the authors conducted the survey for 244 participants, who already have the address. I wonder if the authors can include the positive leptospirosis cases to identify the environmental risk rather than describe the characteristics. These results can support the discussion (lines: 338-347)**

**Response:** As there were very few leptospirosis positive individuals that answered the perception questionnaire, we felt that providing the environmental characteristics of where they lived was of limited use and could risk exposing their identity. Instead, we have included the environmental characteristics of all leptospirosis positive households in each of the study areas, which also contributes to the discussion (see Table 2 and lines 246-249 and 267-270).

**Reviewer #2:** This is an interesting piece of research. The manuscript is well written, and the authors do a good job of presenting the rationale, methods, results, and conclusions. However, there are some weaknesses. The methods need more clarity, some additional statistics is needed, and the discussion should be developed further. See specific comments below.

**Comment 1. Line 107: Change Familiar Income to Family Income in Figure 1.**

**Response:** Thank you for your comment and suggestion. We have implemented the change from “Familiar Income” to “Family Income” in Figure 1.

**Comment 2. Line 117: There are over 250 serovars of Leptospira in the word. The use of only 2 serovars in this study is not addressed.**

**Response:** We thank the reviewer for this valuable observation. The initial version of the manuscript referred to two serogroups (Icterohaemorrhagiae and Cynopteri) because they were the most epidemiologically relevant and most frequently detected in our data. However, we acknowledge that this could have led to the impression that only two antigens were tested.

In fact, the Microscopic Agglutination Test (MAT) panel included seven Leptospira strains, representing a combination of international reference antigens and locally circulating isolates. The two serogroups emphasized in the results (Icterohaemorrhagiae and Cynopteri) were highlighted because they are the predominant infecting serogroups in the study region, in agreement with previous epidemiological surveillance (Khalil [1]; De Oliveira [20]).

We have revised the Methods section to clearly specify all seven strains tested and to explain that the focus on these two serogroups was based on their higher frequency in the study population (Lines 115–131).

**Comment 3. Line 140: Append the questionnaire.**

**Response:** Thank you for your comment. The questionnaire was made available as an appendix in the supplementary materials.

**Comment 4. Line 177: Add a section in methods that identifies all** **software and packages used and cite these.**

**Response:** Thank you for your comment and suggestion. We have included a section in the Methods section entitled “Software and packages,” listing all the software and packages used and citing them (lines 193 -204).

**Comment 5. Line 136: What is the sampling frame? How were the 244 households chosen?**

**Response:** Thank you for your comment and the opportunity to clarify this. The answer to this question is available in comment 3 for Reviewer #1, and we are also providing it here. To define the participants in our study, we used a census sample from the serological survey conducted in the study areas and approached those who were eligible to participate. Eligible residents were individuals aged >18 years or older, who slept three nights per week within the study area, were able to provide written informed consent, and were considered heads of household. We included these details in the manuscript (lines 150-153).

**Comment 6. Results: The entire results section rely heavily on descriptive percentages, tests on significance is needed to make conclusions.**

**Response:** We thank reviewers for highlighting our oversight. We have included p values in Table 4 to be able to fully interpret the Kappa scores presented (see Table 4). However, given that for the other tables we are only presenting descriptive data, we feel it is not appropriate to show p values here. We are not performing any hypothesis testing on these data. Instead, we are using this data to describe our study sample. Therefore, we decided not to present any p values as these would be difficult to interpret, given no hypothesis testing is being carried out. We have followed recommendations given by the ASA statement on p values (Wasserstein, R. L., & Lazar, N. A. (2016). The ASA Statement on *p*-Values: Context, Process, and Purpose.) and those included in Greenland et al 2016 (Greenland S, Senn SJ, Rothman KJ, Carlin JB, Poole C, Goodman SN, Altman DG. Statistical tests, P values, confidence intervals, and power: a guide to misinterpretations. Eur J Epidemiol. 2016).

**Comment 7. Lines 276-287: Should be moved to the methods section I indicated in point 4.**

**Response:** Thank you for your comment and suggestion. We have moved the mentioned information to the methods section (lines 193-204).

**Comment 8. Line 309-314: Apart from vegetation, impervious terrain has been found to be significant risk factor for leptospirosis in urban environments as they flood more readily. Why was perceptions on flooding not considered in this study? In addition, if leptospirosis is mainly a disease of males and most of the participants in this study were females, the results would reflect the risk perceptions of females which may be different to males based on their roles and activities in the community.**

**Response:** Thank you for your comment and the opportunity to clarify this information. In our study, since the risks addressed were a result of what the participants perceived in their communities, and the category of flooding was not mentioned in the responses, it did not appear in the results presented. Furthermore, as mentioned in the comment, our sample was primarily composed of women, and flooding is likely not one of the risks most perceived in the communities by these participants, which may be a result of the lower exposure of people of this gender to risk factors for leptospirosis.

To explore these issues further, we have included some additional information in the discussion section (lines 359-362).

**Comment 9. Line 314-317: I am not sure what this statement is supposed to convey.**

**Response:** Thank you for your comment and the opportunity to also clarify this information. The sentence mentioned presents the peri-domestic environmental characteristics of the group of households included in our research in comparison with the set of households existing in other areas of the communities. Please see the details included in the manuscript (line 335).

**Comment 10. Line 324-325: How was your sample similar to the prevalence study. Was is the number of males and females? Because this appears to be a confounder that needs to be discussed**.

**Response:** Thank you for your comment. In our study we selected participants based on similar inclusion criteria than in the prevalence study. Furthermore, the percentage of female participants, as in our study, was higher than that of men in all communities, ranging from 56.5% in AC and RS to 59.5% in MR, as described in Khalil [1]. We do not believe this presents a confounding factor for our analysis.

**Comment 11. Line 325-327: Is this outlier significant?**

**Response:** Thank you for highlighting the lack of context in this sentence. We have added another sentence to clarify the significance of the high prevalence in RS.

**“**This may have resulted in a higher awareness of the risks associated to leptospirosis and could have biased the results.” (Lines 375-376)

**Comment 12. Line 339-341: Was this difference significant?**

**Response:** Thank you for highlighting the confusing use of vocabulary. We have edited this section to clarify the point we are trying to make.

“In this study, we found that residences located in the AC, MR, and RS communities were surrounded by more vegetation and soil but less impermeable terrain compared to the community average. This difference could offer a more favourable environment for the presence of rodents and could lead to increased contact with soil contaminated with urine from these hosts.” (lines 380 – 384)

**Comment 13. Line 344-346: As mentioned in point 8, impermeable terrain are high-risk flood areas and floods are a risk factor for leptospirosis. Is the prevalence of leptospirosis known in these regions?**

**Response:** In our study areas, the land cover is very heterogeneous and changes swiftly as you move across the neighbourhood. They are patchworks of small adjacent areas of impervious land, vegetation and exposed soil. Therefore, it would be nearly impossible to accurately describe the unique prevalence of impervious land.

**Comment 14. Line 357-358: Any hypothesis as to why more rat positive areas were perceived as low risk for rats?**

**Response:** Thank you for your comment and for asking about this important issue. For us, it is likely that, given the multiplicity of risks to which the population is exposed, some problems become “naturalized”, leading to an underestimation of the presence in specific contexts, especially those further from homes or less visible. Furthermore, although residents recognize the risks associated with rats, the perception of risk appears to be more closely linked to the visibility of the problem, such as the accumulation of garbage, and its concentration in some regions of the community. Unlike garbage, rats do not settle in specific locations; they move according to the availability of food and avoid areas where they perceive danger, which makes their stable identification difficult and may contribute to the underestimation of that risk.

We provide further details about our hypothesis in the manuscript (lines 411-418) and below:

“Here, we hypothesize that this may be related to the multiplicity of risks to which the population is exposed, which can contribute to some problems becoming "naturalized," leading to an underestimation of their presence in specific contexts, especially those further from homes or less visible. In the case of rats, the perception of low risk may be related to the fact that they do not settle in specific locations; they move according to the availability of food and avoid areas where they perceive danger, which makes their stable identification difficult and may contribute to the underestimation of that risk.”

**Comment 15. Lines 361-362: AC was also the region with the highest proportion of females. See comment 8 regarding disease perception and sex.**

**Response:** Thank you for your comment. Please see our response regarding disease perception and sex in comment 8.

**Comment 16. Lines 386-399: It is great to see these 2 paragraphs. Important points that need to be considered in such studies in the future.**

**Response:** Thank you so much for that encouraging comment. We are pleased about that.
